# The burden and predictors of latent tuberculosis infection among immigrants in South Korea: a retrospective cross-sectional study

**DOI:** 10.1186/s12879-021-06922-x

**Published:** 2021-12-03

**Authors:** Sarah Yu, Dawoon Jeong, Hongjo Choi

**Affiliations:** 1 Division of Health Policy, Research Center, The Korean Institute of Tuberculosis, Cheongju, Republic of Korea; 2 School of Health Policy and Management, College of Health Science, Korea University, Seoul, Republic of Korea; 3 Transdiciplinary Major in Learning Health Systems, Department of Healthcare Sciences, Graduate School, Korea University, Seoul, Republic of Korea; 4 Department of Preventive Medicine, Konyang University College of Medicine, Daejeon, Republic of Korea

**Keywords:** Tuberculosis screening, Immigrant screening, Standardized prevalence ratio

## Abstract

**Background:**

Approximately one-fourth of the global population is latently infected with *Mycobacterium tuberculosis*. An understanding of the burden of latent tuberculosis infection (LTBI) among immigrants compared with the general Korean population should be the first step in identifying priority groups for LTBI diagnosis and treatment. The study aimed to compute the age-standardized LTBI prevalence and predictors among immigrants with LTBI in South Korea.

**Methods:**

In 2018, the Korea Disease Control and Prevention Agency implemented a pilot LTBI screening project for immigrants using a chest radiography and the QuantiFERON Gold In-Tube assay. A standardized prevalence ratio (SPR) was computed to compare the LTBI burden in immigrants and the general Korean population.

**Results:**

During the duration of the project, a total of 8108 immigrants (5134 males and 2974 females) underwent LTBI screening. The SPR of 1.547 (95% confidence interval [CI] 1.468–1.629) in males and 1.261 (95% CI 1.177–1.349) in females were both higher than the Korean reference population. Furthermore, among the immigrants, those aged < 40 years and Korean diaspora visa holders had a higher SPR.

**Conclusion:**

This study found a higher LTBI prevalence among immigrant population in South Korea compared to that in the general Korean population, and the SPR was higher among those aged < 40 years and the Korean diaspora. The findings can be used as baseline evidence for including immigrants in South Korea in the at-risk group with a priority need for LTBI screening and treatment.

**Supplementary Information:**

The online version contains supplementary material available at 10.1186/s12879-021-06922-x.

## Background

The global burden of latent tuberculosis infection (LTBI) is estimated to be approximately one fourth of the global population [1–3] LTBI is defined as a state of infection with *Mycobacterium tuberculosis* (MTB) without symptoms and infectivity, however it is still a major topic of interest in public health because it can progress to active tuberculosis (TB) [4]. Therefore, LTBI management, including testing and treatment, is one of the key components of the End TB Strategy by the World Health Organization (WHO) [5]. Not all people with LTBI necessarily progress to active TB; thus, screening the high-risk group who require LTBI testing and treatment is crucial.

The WHO Guideline Development Group (GDG), through a systematic review, has identified high-risk groups that need LTBI testing and treatment based on the LTBI infection rate, the possibility of progression to active TB, and burden of active TB, compared to those in the general population, as well as the risk and benefits of examination and treatment [6]. Based on these results, the GDG proposes people living with human immunodeficiency virus (HIV) and children < 5 years old who are household contacts of people with infectious TB as the at-risk groups with the highest priority for LTBI screening and treatment. In addition to the clinical characteristics, the GDG proposes certain groups of people to have intermediate priority, and these groups include prisoners, health workers, the homeless, drug abusers, and immigrants from high TB burden countries [6].

The priority groups for LTBI testing and treatment have a higher prevalence of LTBI and risk of progression to active TB than the general population. Compared to the LTBI burden in the general population globally (23.0%) [2], the prevalence of LTBI among health care workers in low/middle-income countries was reported to be 49% with the tuberculin skin test and 39% with the interferon γrelease assay in a systematic review [7]. Furthermore, the risk of progression to active TB is higher in younger children (15% within 5 years of age; up to 33% within 1 year of age) [8], HIV co-infected (26-fold higher risk of TB reactivation) [9], and immigrants from countries with a high incidence of TB (≥ 40 cases per 100,000 population) (relative risk 30.7 with 95% confidence interval [CI] 17.5–54.1 within 12 months) [10] than those in the general population. Moreover, 5–10% of these groups may progress to active TB in their lifetime [11].

Providing targeted LTBI screening and treatment to immigrants in high-income, low TB burden countries was particularly emphasized because of migration from low/middle-income high TB burden countries [12]. Industrialized countries have shared a similar TB epidemiological shift with decreasing incidence in the native-born but increasing incidence in the foreign-born [13]. Among the 30 low burden countries, over 50% of all notified TB cases in 2015 were foreign-born [14]. One third of all new active TB cases were diagnosed in people who were born in other countries among European countries in 2016 [15]. However, countries with low TB burden make their decisions based on different epidemiologic situation, and they may have similar LTBI prevalence among immigrants compared to the general population. Thus, context-based evidence are required to prioritize a specific risk group for LTBI screening and treatment.

South Korea is a high-income country with an intermediate TB burden, and LTBI screening in a large population showed a positivity rate of 19.0% [16]. The priority groups in need of LTBI treatment differ slightly in the Korean guideline for TB, published jointly by the Korean TB society and the Korea Disease Control and Prevention Agency (KDCA) [17], compared to the GDG. Although the ranking of priority groups according to their clinical characteristics is consistent with the WHO recommendations, the Korean guideline does not include recommendations for specific groups, i.e., immigrants and the homeless [18]. Korea’s history of rapid economic growth highlights the importance of LTBI control for immigrants; however, there has not been an appropriate analysis of the LTBI risks of immigrants compared to the general population in Korea. Therefore, the Korean guideline also does not contain this relevant information [19].

The aim of this study was to compute the age-standardized LTBI prevalence and predictors of LTBI among immigrants in South Korea to examine their LTBI burden and risk factors.

## Methods

### Study population, setting, and design

In this retrospective cross-sectional study, data from a pilot LTBI screening project for immigrants were used, which was planned by the KDCA and implemented by the Korean National Tuberculosis Association in 2018. The Korean government requires (1) immigrants from a country with a TB burden of 50 per 100,000 population to undergo pre-entry active TB screening when they apply for a visa granting stay in South Korea for 91 days or longer and (2) immigrants from a country with a high TB risk to undergo TB screening when they change or renew their visa while residing in South Korea. The KDCA project was targeted at the immigrants who need to renew their visa status, and chest radiography and QuantiFERON Gold In-Tube assays (QFT-GIT; Qiagen, Hilden, Germany) were performed [20]. The pilot project was conducted from August 21, 2018, to December 21, 2018, in Gyeonggi Province, which has a population of 13.4 million and the highest foreign immigrant population in South Korea (410 000 out of 1.3 million).

### Measurement and statistical analysis

The data obtained from the pilot project were analyzed after removing all personal identifiable information. We included participants who had normal chest X-ray in the analysis. The variables included age (< 20, 20–29, 30–39, 40–49, 50–59, ≥ 60 years), sex (male, female), the disease burden of the country of origin (high burden and low-intermediate burden countries), TB history (yes, no), age at entry into South Korea (< 20, 20–29, 30–39, 40–49, 50–59, ≥ 60 years), length of stay in South Korea (< 1, 1–4, ≥ 5 years), and visa type (Korean diaspora and others). It is noteworthy that a substantial percentage of immigrants in South Korea are Korean diaspora, who are the subsequent generations of Korean people who had immigrated to foreign countries since the 1860s. Understanding this group is crucial to identifying the characteristics of immigrants in South Korea [21].

The participants’ demographic characteristics were compared using Pearson’s chi-squared tests. A standardized prevalence ratio (SPR) was computed to compare the LTBI burden in immigrants with that of the general Korean population. Further, the study by Hwang et al. (2018), which reported screening of the largest group of people so far, was used as a reference for the age-specific LTBI burden in the Korean population. The equations for the SPR and 95% CI are shown below [22].

$$SPR = \frac{\sum Observed \, number \, of \, LTBI}{{\sum Expected \, number \, of LTBI}} = \frac{{\mathop \sum \nolimits_{i} d_{i} }}{{\mathop \sum \nolimits_{i} t_{i} \left( {\frac{{D_{i} }}{{T_{i} }}} \right)}},$$

$$95 \% \,confidence\, interval \left( {CI} \right) = \frac{SPR}{{exp\left( {\frac{1.96}{{\sqrt d }}} \right)}} to \,SPR \times exp\left( {\frac{1.96}{{\sqrt d }}} \right).$$

where $i =$ 10-year age group (< 20, 20–29, 30–39, 40–49, 50–59, ≥ 60 years), $T_{i} =$ age-specific population of the general population, $D_{i} =$ age-specific number of LTBI of the general population, $t_{i} =$ age-specific population of immigrant participants, and $d_{i} =$ age-specific number of LTBI of immigrant participants. Additionally, we analyzed the differences in SPR according to age, sex, and visa type. Finally, sex-specific multivariate logistic regression models were conducted to identify the predictors of LTBI in immigrants. Microsoft PowerPoint and Excel [Microsoft 365, version 2108] were used to create plot diagrams.

## Results

During the duration of the project, a total of 8205 immigrants participated in LTBI screening. We excluded immigrants who had abnormal chest X-ray findings (n = 97); these were referred to the National TB program for further evaluation. Finally, in total, 8108 immigrants were included in the analysis, who included 5134 male and 2974 female immigrants. The prevalence of LTBI was 28.1% (95% CI 27.1–29.1), which was significantly higher than that in the reference Korean population (19.0%; 95% CI 18.5–19.5) [16]. In both the male and female immigrant groups, the LTBI prevalence was significantly higher with increasing age, TB burden of the country of origin, visa type for overseas Koreans, TB history, older age at entry into South Korea, and longer stay in South Korea (Table 1, Additional file 1).

**Table 1:** Sex-stratified distribution of latent tuberculosis infection by baseline characteristics (n = 8108)

Variable	Total number of LTBI (n = 8108)			Male (n = 5134)			Female (n = 2974)		
	n	%	*P* value	n	%	*P* value	n	%	*P* value
Total	2275	28.06		1434	27.93		841	28.28	
Age group (years)			< 0.001			< 0.001			< 0.001
< 20	11	6.04		9	8.74		2	2.53	
20–29	267	14.47		195	14.72		72	13.85	
30–39	473	21.00		348	21.25		125	20.36	
40–49	536	34.08		339	35.87		197	31.37	
50–59	766	42.89		435	47.96		331	37.66	
60+	222	47.23		108	50.00		114	44.88	
Country of origin			< 0.001			< 0.001			< 0.001
Low-intermediate TB burden	377	19.41		249	19.48		128	19.28	
High TB burden	1898	30.78		1185	30.73		713	30.87	
Visa type			< 0.001			< 0.001			< 0.001
Korean diaspora	1794	33.00		1092	34.71		702	30.66	
Others	481	18.00		342	17.20		139	20.32	
History of TB			< 0.001			< 0.001			0.028
No	2231	27.82		1405	27.67		826	28.09	
Yes	44	49.44		29	51.79		15	45.45	
Age at entrance (years)			< 0.001			< 0.001			< 0.001
< 20	34	9.86		27	12.68		7	5.30	
20–29	499	18.52		370	19.03		129	17.18	
30–39	516	24.93		357	24.54		159	25.85	
40–49	637	38.56		373	41.82		264	34.74	
50–59	478	43.73		258	49.33		220	38.6	
60+	111	43.87		49	45.79		62	42.47	
Duration of stay (years)			< 0.001			< 0.001			< 0.001
< 1	729	24.91		431	25.34		298	24.31	
1–4	678	24.84		455	23.16		223	29.19	
5+	868	35.40		548	37.33		320	32.52	

LTBI, latent tuberculosis infection with QuantiFERON Gold In-Tube positive and chest X-ray negative; TB, tuberculosis

The SPR was 1.55 (95% CI 1.47–1.63) in males and 1.26 (95% CI 1.18–1.35) in females, both of which were higher than those of the Korean reference population. However, in contrast to the crude LTBI prevalence, the SPR was higher among those aged < 40 years than the ≥ 40 years group, in both male and female immigrants (< 40 years: 1.72, 95% CI 1.60–1.85 ≥ 40 years: 1.32, 95% CI 1.253–1.38). The SPR did not vary greatly according to the country of origin but was higher among those from a country with a high TB burden, and the higher SPR according to those with a history of TB was also consistent between the male and female groups. Although the SPR was higher among male Korean diaspora, the difference was less among the females. Further, the SPR increased with the increasing length of stay in South Korea in male immigrants, but the SPR was highest in female immigrants with 1–4 years of stay in South Korea (Table 2).

**Table 2:** Sex-stratified age-standardized prevalence ratio and NNS (number needed to screen) by covariates of latent tuberculosis infection

Variable	Total				Male				Female			
	SPR	95% CI		NNS	SPR	95% CI		NNS	SPR	95% CI		NNS
Total	1.43	1.37	1.49	4	1.55	1.47	1.63	4	1.26	1.18	1.35	4
Age (years)												
< 40	1.72	1.60	1.85	6	1.75	1.61	1.91	6	1.65	1.43	1.89	6
40+	1.32	1.25	1.38	3	1.44	1.35	1.54	2	1.18	1.09	1.27	3
Country of origin												
High TB burden	1.44	1.38	1.51	3	1.56	1.47	1.65	3	1.28	1.19	1.38	3
Low-intermediate TB burden	1.35	1.21	1.49	5	1.48	1.30	1.67	5	1.15	0.96	1.36	5
Visa type												
Korean diaspora	1.46	1.40	1.53	3	1.63	1.53	1.73	3	1.27	1.17	1.36	3
Others	1.31	1.19	1.43	6	1.33	1.20	1.48	6	1.24	1.04	1.46	5
TB history												
No	1.42	1.36	1.48	4	1.53	1.45	1.62	4	1.25	1.17	1.34	4
Yes	2.30	1.67	3.09	2	2.74	1.83	3.93	2	1.79	1.00	2.95	2
Duration of stay (years)												
< 1	1.39	1.29	1.49	4	1.53	1.39	1.68	4	1.25	1.11	1.39	4
1–4	1.46	1.35	1.57	4	1.54	1.40	1.68	4	1.32	1.15	1.51	3
≥ 5	1.44	1.35	1.54	3	1.57	1.44	1.71	3	1.26	1.13	1.41	3

LTBI, latent tuberculosis infection with QuantiFERON Gold In-Tube QFT-GIT positive and chest X-ray (CXR) negative

SPR, age-standardized prevalence ratio of latent tuberculosis infection; CI, confidence interval; TB, tuberculosis

**Fig. 1:**
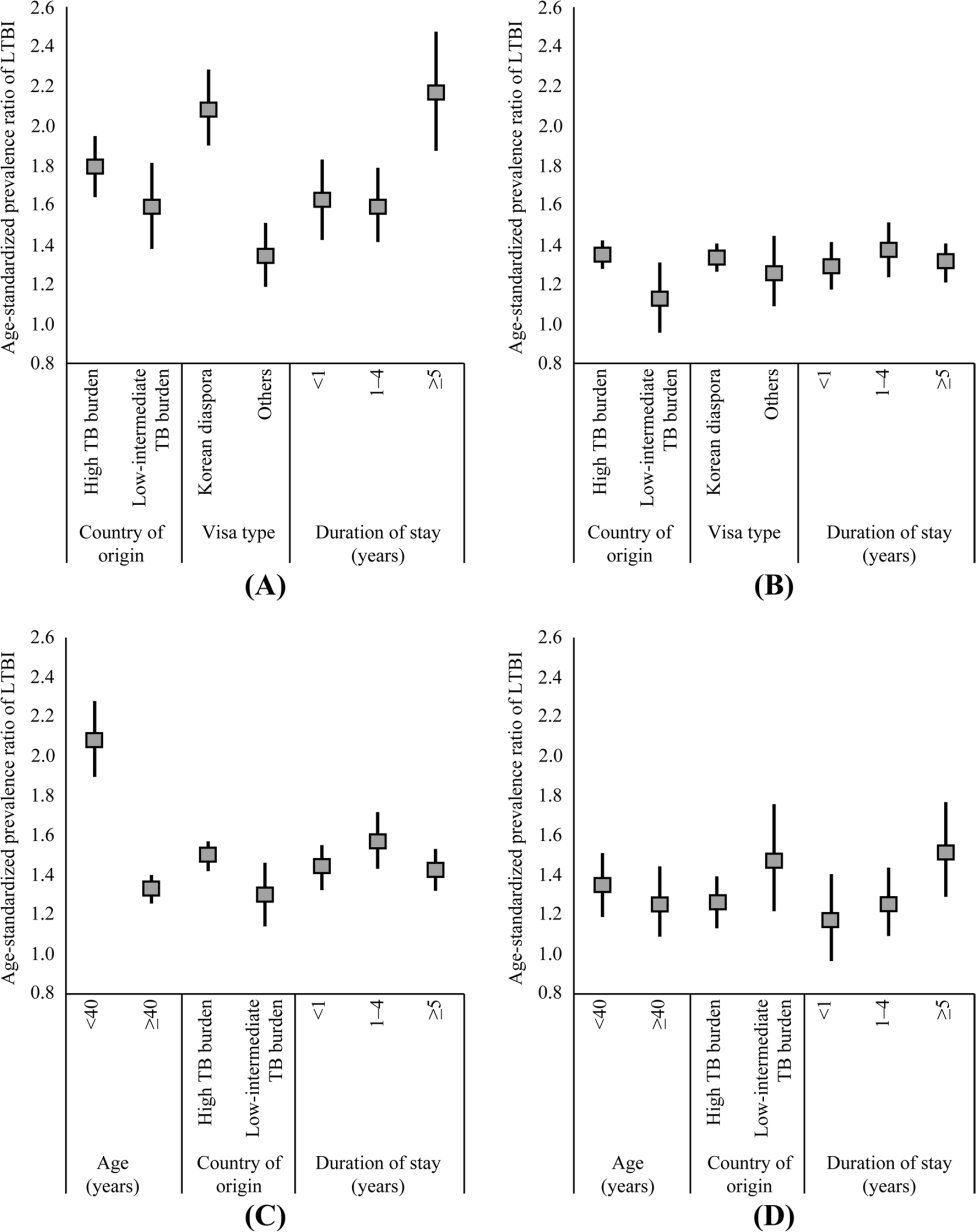
Age-standardized prevalence ratio of latent tuberculosis infection within age strata and visa type. **A** < 40 years old; **B** ≥ 40 years old; **C** Korean diaspora; **D** other visa type. The box shows the age-standardized prevalence ratio, and the vertical lines represent the 95% confidence intervals. Variables: country of origin (high TB burden and low-intermediate TB burden), visa type (Korean diaspora and others), duration of stay (< 1 year, 1–4 years, and ≥ 5 years), and age (< 40 and ≥ 40 years). TB = tuberculosis; LTBI = latent tuberculosis infection

The differences in the SPR according to age (< 40 and ≥ 40 years) and Korean diaspora were additionally analyzed (Fig. 1). The SPR was highest for the Korean diaspora < 40 years (2.1, 95% CI 1.9–2.3) and for immigrants aged < 40 years who had stayed in South Korea for ≥ 5 years (2.2, 95% CI 1.9–2.5). In addition, the gap in the SPR according to the length of stay in South Korea substantially decreased among the Korean diaspora, with the SPR ranging from 1.4–1.6, regardless of the length of stay in South Korea.

Finally, in a multivariate analysis, the LTBI prevalence was found to be associated with the male sex, older age, TB burden of the country of origin, Korean diaspora, TB history, older age at entry into South Korea, and longer stay in South Korea (Table 3).

**Table 3:** Predictors of latent tuberculosis infection among immigrants in South Korea

Variable	Total						Male						Female					
	Univariate			Multivariate			Univariate			Multivariate			Univariate			Multivariate		
	OR	95% CI		OR	95% CI		OR	95% CI		OR	95% CI		OR	95% CI		OR	95% CI	
Sex																		
Male	1			1														
Female	1.02	0.92	1.12	0.77	0.70	0.86												
Age (years)																		
< 40	1			1			1			1			1			1		
40+	3.11	2.80	3.44	1.68	1.37	2.07	3.39	2.98	3.84	1.70	1.32	2.18	2.92	2.44	3.50	1.55	1.08	2.23
Country of origin																		
High TB burden	1.85	1.63	2.09	1.30	1.12	1.49	1.83	1.57	2.14	1.22	1.02	1.46	1.87	1.51	2.31	1.42	1.11	1.81
Low-intermediate TB burden	1			1			1			1			1			1		
Visa type																		
Korean diaspora	2.24	2.00	2.51	1.54	1.36	1.75	2.56	2.23	2.94	1.68	1.43	1.96	1.73	1.41	2.13	1.24	0.99	1.55
Others	1			1			1			1			1			1		
TB history																		
No	1			1			1			1			1			1		
Yes	2.54	1.67	3.85	2.49	1.61	3.85	2.81	1.66	4.76	2.93	1.68	5.11	2.13	1.07	4.25	1.87	0.92	3.80
Age at entry (years)																		
< 20	1			1			1			1			1			1		
20–29	2.08	1.44	3.00	1.93	1.33	2.80	1.62	1.06	2.46	1.61	1.05	2.46	3.70	1.69	8.12	3.43	1.56	7.54
30–39	3.04	2.10	4.39	2.15	1.47	3.15	2.24	1.47	3.41	1.69	1.09	2.61	6.23	2.85	13.61	4.56	2.04	10.20
40–49	5.74	3.97	8.29	2.72	1.79	4.14	4.95	3.24	7.57	2.27	1.39	3.70	9.50	4.38	20.65	5.20	2.21	12.25
50–59	7.11	4.89	10.33	3.75	2.44	5.77	6.71	4.33	10.40	3.32	1.99	5.54	11.22	5.15	24.48	6.62	2.78	15.80
60+	7.15	4.64	11.02	3.61	2.23	5.86	5.82	3.33	10.13	2.74	1.49	5.06	13.18	5.75	30.20	7.28	2.92	18.17
Duration of stay (years)																		
< 1	1			1			1			1			1			1		
1–4	1.00	0.88	1.12	1.13	0.98	1.29	0.89	0.76	1.03	1.16	0.98	1.38	1.28	1.05	1.57	1.14	0.91	1.43
≥ 5	1.65	1.47	1.86	1.41	1.22	1.64	1.76	1.51	2.04	1.51	1.25	1.82	1.50	1.25	1.81	1.29	1.01	1.63

OR, odds ratio; CI, confidence interval; TB, tuberculosis

## Discussion

This study aimed to compute the ratio of LTBI prevalence in particular groups to that of the general population, and the key evidence needed to determine priority for LTBI diagnosis and treatment among immigrants in South Korea. The main findings are described below. First, we confirmed that immigrants have a higher LTBI SPR compared to the general Korean population. Furthermore, among the immigrants, those aged < 40 years and Korean diaspora had a higher SPR.

Similar findings have been reported in other high-income countries. A large-scale pre-entry and post-entry TB screening report in the United Kingdom showed that the LTBI risk was about 1.39 times higher among the younger age group (16–25 years) compared to the older age group (26–35 years), and the LTBI prevalence was higher among settlements and dependents or family reunion visa holders compared to student visa holders [23]. A large-scale US study that evaluated differences in the LTBI diagnostic methods also reported a similar LTBI prevalence between recent TB contacts and recent immigrants. This suggests that recent TB contacts and immigrants have a similar LTBI burden to recent TB contacts who are considered to be a priority for LTBI screening and treatment [24]. A Japanese study also reported that immigrants from a few countries showed a high LTBI prevalence [25].

However, there are a few challenges to providing LTBI screening and treatment for all immigrants in many countries; one prime example is the evidence of its cost-effectiveness. A study on LTBI in immigrants in Europe reported the paucity of data on the cost-effectiveness of LTBI screening and treatment for the entire immigrant population, but reported data supporting the cost-effectiveness for young immigrants [26]. Moreover, recent studies have emphasized the importance of priority determination based on risks because even if immigrants from high TB burden countries show a high LTBI prevalence, the cost-effectiveness of implementing a universal screening and treatment strategy for all such immigrants remains controversial [27]. Furthermore, considering the high mobility of immigrants, the risk for premature LTBI treatment termination also needs to be considered [28]. Many immigrants in high-income countries are socio-economically vulnerable and suffer from the difficulties arising from cultural gaps, and this should be taken into consideration when devising LTBI strategies [14]. The vulnerability may be an barriers to start and continue LTBI treatment and almost 10% of LTBI immigrants agreed to receive LTBI treatment and about 60% completed the therapy in our study setting [20].

This study has a few limitations. First, it was not easy to compare the LTBI prevalence between immigrants in South Korea and Korean nationals due to the lack of accurate data on the age-specific LTBI prevalence in the Korean population. However, to overcome this limitation, we used the data on the age-specific LTBI prevalence of a general population with a relatively low risk of active TB, as opposed to using the LTBI prevalence data from a group with a high risk of active TB, such as recent TB contacts. Second, the fact that the study participants were immigrants living in a single region who needed visa extension or renewal limits the representativeness of the sample. However, as the pilot study was conducted in a region with the highest immigrant population in South Korea and included immigrants of various ages and visa types, it was possible to examine the trend of LTBI prevalence among immigrants. Finally, this study only presented data on the LTBI prevalence compared to that of the general population; this is only one of the three key factors (the LTBI prevalence, the possibility of progression to active TB, and burden of active TB), needed to determine the priority of LTBI screening and treatment [6]. Thus, these findings form part of the evidence needed to determine the priorities for LTBI screening and treatment among immigrants. Considering the continuously growing immigrant population in South Korea, the steady number of foreigners diagnosed with TB, and the high active TB burden among immigrants of other high-income countries, we can speculate that immigrants in South Korea may have a large burden of active TB [19]. To overcome this limitation, future studies should focus on comparing the incidence of active TB in the Korean and immigrant populations, and examine the risk factors for the progression of LTBI to active TB.

## Conclusion

This study found that the immigrant population in South Korea had a high LTBI prevalence compared to the general Korean population and that the SPR was higher among those aged < 40 years and the Korean diaspora. Within the scope of available resources, the Korean government should develop a pilot project or study on LTBI screening and treatment to determine its potential in preventing active TB in the Korean diaspora aged < 40 years.

## Supplementary Information

**Additional file 1:** The prevalence of LTBI according to the nationality of immigrants.

## Data Availability

The datasets generated and/or analyzed during the current study are available from the corresponding author on reasonable request.

## Abbreviations

CI: Confidence interval
GDG: WHO Guideline Development Group
HIV: Human immunodeficiency virus
KDCA: Korea Disease Control and Prevention Agency
KHIDI: Korea Health Industry Development Institute
KYUIRB: Konyang University Institutional Review Board
LTBI: Latent tuberculosis infection
MTB: *Mycobacterium tuberculosis*
QFT-GIT: QuantiFERON Gold In-Tube assays
SPR: Standardized prevalence ratio
TB: Tuberculosis
WHO: World Health Organization
