# Supplementary material for: The burden and predictors of latent tuberculosis infection among immigrants in South Korea: a retrospective cross-sectional study
Source: BMC Infect Dis. 2021 Dec 3;21:1206. doi: 10.1186/s12879-021-06922-x (PMC8641149; doi:10.1186/s12879-021-06922-x)
Supplement: Supplementary file 1 — Additional file 1: The prevalence of LTBI according to the nationality of immigrants. [file 12879_2021_6922_MOESM1_ESM.docx]

Table S1 The prevalence of LTBI according to the nationality of immigrants

| **High TB burden Country** | | | | **Low-intermediate TB burden country** | | | |
| --- | --- | --- | --- | --- | --- | --- | --- |
| Country | Total | LTBI Positive | % | Country | Total | LTBI Positive | % |
| Bangladesh | 107 | 17 | 15.9 | Australia | 1 | 0 | 0.0 |
| Cameroon | 1 | 1 | 100.0 | Bhutan | 1 | 0 | 0.0 |
| China | 4,659 | 1,697 | 36.4 | Canada | 7 | 0 | 0.0 |
| Indonesia | 150 | 17 | 11.3 | Egypt | 1 | 0 | 0.0 |
| India | 31 | 7 | 22.6 | United Kingdom | 2 | 0 | 0.0 |
| Cambodia | 158 | 11 | 7.0 | Jordan | 1 | 1 | 100.0 |
| Liberia | 1 | 1 | 100.0 | Japan | 5 | 0 | 0.0 |
| Myanmar | 143 | 30 | 21.0 | Kyrgyzstan | 57 | 8 | 14.0 |
| Philippines | 188 | 28 | 14.9 | Kazakhstan | 214 | 43 | 20.1 |
| Russia | 156 | 39 | 25.0 | Sri Lanka | 75 | 4 | 5.3 |
| Thailand | 202 | 15 | 7.4 | Morocco | 3 | 1 | 33.3 |
| Viet Nam | 368 | 35 | 9.5 | Mongolia | 72 | 18 | 25.0 |
| South Africa | 2 | 0 | 0.0 | Malaysia | 4 | 0 | 0.0 |
| **Total** | **6,166** | **1,898** | 30.8 | Nigeria | 1 | 0 | 0.0 |
|  |  |  |  | Nepal | 287 | 67 | 23.3 |
|  |  |  |  | Pakistan | 25 | 3 | 12.0 |
|  |  |  |  | Tajikistan | 8 | 2 | 25.0 |
|  |  |  |  | East Timor | 6 | 2 | 33.3 |
|  |  |  |  | Turkmenistan | 6 | 1 | 16.7 |
|  |  |  |  | Taiwan | 8 | 0 | 0.0 |
|  |  |  |  | Ukraine | 79 | 18 | 22.8 |
|  |  |  |  | United States | 10 | 1 | 10.0 |
|  |  |  |  | Uzbekistan | 1,064 | 208 | 19.5 |
|  |  |  |  | Yemen | 5 | 0 | 0.0 |
|  |  |  |  | **Total** | **1,942** | **377** | 19.4 |

LTBI = latent tuberculosis infection; TB = tuberculosis
